# Does transcatheter aortic valve alignment matter?

**DOI:** 10.1136/openhrt-2019-001132

**Published:** 2019-11-21

**Authors:** Jacob Andrew Salmonsmith, Andrea Ducci, Gaetano Burriesci

**Affiliations:** 1 UCL Mechanical Engineering, University College London, London, UK; 2 Unit of Bioengineering, Ri.MED Foundation, Palermo, Italy

**Keywords:** transcatheter aortic valve, valve alignment, valsalva sinuses

## Abstract

**Objective:**

This study investigates the effect of transcatheter aortic valve (TAV) angular alignment on the postprocedure haemodynamics. TAV implantation has emerged as an effective alternative to surgery when treating valve dysfunction. However, the benefit of avoiding surgery is paid back by the inability to remove the native diseased leaflets and accurately position the device in relation to the aortic root, and the literature has shown the root anatomy and substitute position can play an essential role on valve function.

**Methods:**

A commercial TAV was placed in a silicone mock aortic root in vitro, including mock native leaflets, and either aligned commissure-to-commissure or in maximum misalignment. Haemodynamic performance data at various stroke volumes were measured, and Particle Image Velocimetry analysis was performed at a typical stroke volume for rest conditions. The two configurations were also studied without mock native leaflets, for comparison with previous in vitro studies.

**Results:**

Haemodynamic performance data were similar for all configurations. However, imaging analysis indicated that valve misalignment resulted in the central jet flow not extending to the root wall in the native commissures’ vicinity, replaced by a low shear flow, and a reduction of upper sinus flow of 40%, increasing flow stagnation in the sinus.

**Conclusions:**

TAV misalignment did not result in a significant change in valve hydrodynamic performance, but determined some change in the fluid flow patterns, which may promote pathological scenarios, such as increased thrombogenicity of blood flow within the sinuses of Valsalva, and plaque formation around the lumen of the sinotubular junction.

**Key questions**

**What is already known about this subject?**

- There will be an expected increased use of transcatheter aortic valves (TAVs) due to ageing populations, but the most common products on the market are difficult to rotationally align with the native aortic root’s Valsalva sinuses.

**What does this study add?**

- The alignment of a TAV with its native aortic root does not appear to have any significant impact on the global haemodynamics of the valve. Native to prosthetic commissural alignment may be beneficial to the washout of the sinuses, potentially decreasing thrombogenicity.

**How might this impact on clinical practice?**

- Optimum valve orientation should not be a consideration if it causes an increase to the risk of the procedure. However, as misalignment may reduce sinus flow and create a more thrombotic environment, the link between subclinical thrombosis and non-aligned TAV could be investigated *in vivo*.

## Introduction

While surgical aortic valve (SAV) replacement is the standard-of-care for patients with severe aortic stenosis, the presence of comorbidities for many patients makes the risk of complications and long recovery time too high for surgery.^1 2^ Due to a globally ageing population, the frequency of these comorbidities has experienced a rapid increase in the latest decades and is set to rise substantially in the near future. As a result, alternative treatments to surgery have been developed and are now in common usage. Transcatheter aortic valve (TAV) implantation/replacement is a recent non-surgical approach that has been performed on over 200 000 patients.^3^ The valve implantation is achieved guiding percutaneously a delivery system through the patient’s vasculature using intraoperation 2D imaging techniques, such as angiography or fluoroscopy. However, though these techniques are adequate to provide a sufficiently accurate positioning of the depth of the implantation (axial alignment), they are not ideal to identify the angular position of TAVs, while the use of real-time 3D Computed Tomography-fluoroscopy or 3D transoesophageal echocardiography-fluoroscopy fusion imaging, which would enable rotational alignment of prosthetic and native commissures, is still far from being widespread.^4 5^ As a result, although designed to reproduce the tri-leaflet layout of the aortic valve, implantation of TAVs in the same leaflet-to-sinus arrangement as in the native aortic root is rather unsystematic.^6^ This raises some concern about the potential effect of the angular orientation of the implant on the haemodynamics and fluid flow behaviour, as the interaction of the leaflets and fluid flow with the geometry of the sinuses has been shown in vivo, in vitro and numerically to have significant effects on the haemodynamics.^7–11^ Alignment of the commissures may affect the flow dynamics of the sinuses^6^ and has been shown to minimise the stress experienced by the prosthetic’s leaflets.^12^ A study of the implantation depth and rotation of a self-expanding TAV within a bioprosthetic valve has shown that non-alignment of the TAV may increase shear stress within the sinuses.^13^ The objective of this in vitro study is to investigate the effect of the angular alignment of a TAV within the native anatomy on the hydrodynamics produced in the aortic root and observe whether changes to the rotational orientation of TAVs can result in suboptimal haemodynamics and performance. In addition, as the presence of the native leaflets is commonly neglected for in vitro experiments,^14–17^ it is useful to verify if this assumption is acceptable.

## Methods

The valve selected for this study was an Edwards SAPIEN XT, size 26 mm, a widely implanted TAV device, consisting of a cobalt chromium balloon-expandable cellular frame, hosting a trileaflet bovine pericardial valve.

*In vitro* assessment of the valve was carried out on a hydromechanical pulse duplicator Vivitro Superpump System SP3891 (ViVitro Labs, Canada), with a fluid analogue matching the blood viscosity of 4.0 cP at 37°C, and the refractive index of the silicone material used in the mock aortic root.^18^ Tests were performed at a heart rate of 70 beats per minute, with 35% of systolic duration and a mean aortic pressure of 100 mm Hg, at four different stroke volumes distributed between 28.6 mL and 100 mL (corresponding to cardiac outputs varying from 2 to 7 litres per minute (lpm) respectively).

An optically clear silicone (MED-6015, NuSil Technology, California, USA, refractive index n=1.4) mock aortic root was created with annulus and sinotubular junction (STJ) characterised by a diameter of 25 mm. The geometric proportions were based on the description by Swanson and Clark,^19^ the Valsalva sinuses transversal section was defined as an epitrochoid, according to the profile identified by Reul *et al*,^20^ the leaflet dimensions as defined by Thubrikar *et al*,^21^ and the sagittal plane sinus profile specified by Grigioni *et al*^22^ was used to model the Valsalva sinuses. A thick-wall root with negligible compliance was used, as root elasticity tends to reduce in older patients, who are more prone to senile calcification,^3^ resulting in stiffened aortic roots.^23^

Expanded native leaflets were modelled by including a cylindrical vinyl wrap around the TAV, 0.45 mm thick, with its distal edge 17.5 mm from the sinus base and shape matching a fully open native human aortic valve as described by Thubrikar^24^ for an aortic root with 25 mm STJ.

**Figure 1:**
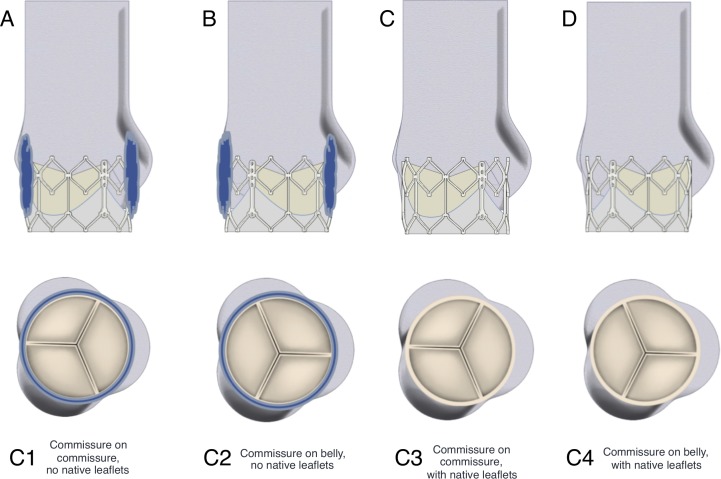
Valve-root configurations considered in study. (A) Configuration C1—commissures of TAV and aortic root model aligned, TAV placed within a cylinder to simulate native leaflets. (B) Configuration C2—TAV 60° out of phase with ideal alignment, native leaflets model again incorporated. (C) Configuration C3—the same valve-root alignment at C1, no native leaflets modelled. (D) The same valve-root alignment at C2, no native leaflets modelled.

The expanded Edwards SAPIEN XT valve was placed into the mock aortic root, with the midpoint of the valve between 1 and 2 mm downstream of the basal annulus of the root,^25^ as recommended for the clinical procedure.^26^ Four different configurations were analysed in this study, as illustrated in figure 1:

C1. Aligned, native leaflets: each commissure of the TAV aligns with a commissure of the aortic root model, resulting in each TAV leaflet’s belly opening out into its associated Valsalva sinus; mock native leaflets were included in the aortic root model.

C2. Misaligned, native leaflets: valve is 60° out of phase with the ideal alignment with the root, resulting in the belly of each TAV leaflet aligning with a commissure of the aortic root model; mock native leaflets were included in the aortic root model.

C3. Aligned, no native leaflets: the same valve-root alignment as used in C1; no native leaflets are included in the model.

C4. Misaligned, no native leaflets: the same valve-root alignment as used in C2; no native leaflets are included in the model.

**Figure 2:**
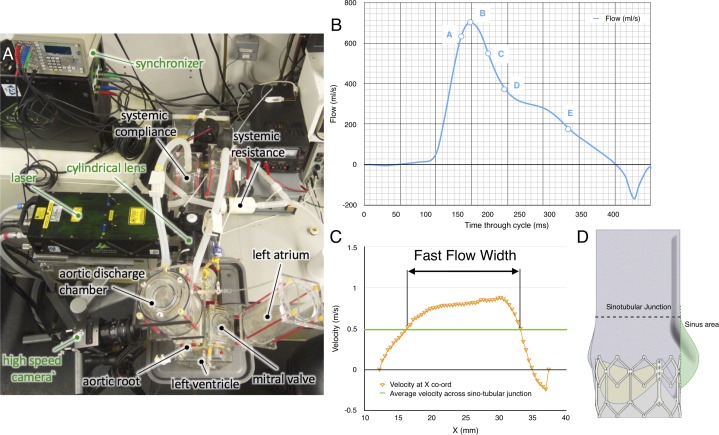
Particle image velocimetry measurement details. (A) Top view of the system setup. (B) Typical diagram of the flowrate versus time during systolic phase, with the instants analysed labelled A–E. (C) Diagram of the sinus area within cross-section image, with the upper half of the sinus indicated by red shading and the lower half by orange shading. (D) Example of fast flow width measurement.

2D Particle image velocimetry (PIV), a laser-based, non-intrusive optical technique, was used to investigate instantaneous fluid dynamics of each configuration at a standard stroke volume of 71.4 mL/cycle (corresponding to a systolic cardiac output of 5 lpm), to produce a vector map describing the instantaneous fluid motion across the measurement plane. The positions of the camera and laser with respect to the valve-root configuration are described in figure 2A. The laser sheet was projected over the sagittal plane, at the centre of the root-valve configuration.

The PIV data were analysed using a phase-resolved approach, averaging the resultant vector maps from a particular reference instant over 150 cycles. After synchronisation of the camera, laser and pulse duplicator, five reference instants were considered for comparison (see figure 2B), corresponding to the times when the ejected flow measured from the flowmeter reached the following conditions:

1. flow increased to 90% of peak flow;
2. peak flow;
3. flow reduced to 75% of peak flow;
4. flow reduced to 50% of peak flow;
5. flow reduced to 25% of peak flow.

The peak velocity, *v_p_,* was identified as the highest velocity magnitude recorded in the region of investigation for each flow condition, A–E. The average sinus velocity, *v_SI_*, was calculated by averaging the velocity magnitudes of any vectors within the sinus area of the PIV cross-sectional image, providing a broad but quantified configuration-to-configuration comparison. The sinus area is indicated as the shaded region in figure 2C with the upper half of the sinus indicated by red shading and the lower half by orange shading. The average velocity magnitude for the upper sinus, *v_USI_*, and the lower sinus, *v_LSI_*, was also calculated to provide a more detailed analysis of the flow dynamics within the sinus throughout systole, to reveal whether the alignment and native leaflet changes affect the flow closer to the STJ or the base of the sinuses to a greater or lesser extent.

In order to be able to quantitatively measure and compare the central jet flow width resulting from each configuration, the fast flow width (FFW) of the flow was defined as the width of the cross section at the STJ where the velocity magnitude, *v*, is higher than 1/3 of *v_p_* measured from the PIV data at peak flow (Instant B) for that cross section (see figure 2D).

Further information on the PIV settings and the fluid properties derived from the PIV data are presented in the appendices.

## Results

**Figure 3:**
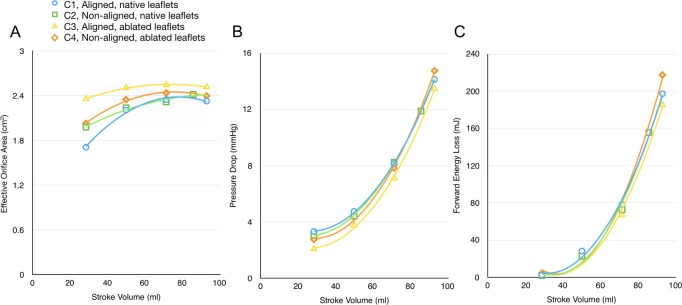
Valve performance during forward flow for each configuration at each stroke volume. (A) Effective orifice area. (B) Transaortic pressure drop. (C) Transaortic energy losses.

The global hydrodynamic performance determined for all configurations are summarised in table 1, which reports the effective orifice area (EOA), the mean transvalvular systolic pressure drop (∆p) and the forward flow energy losses. Results indicate satisfactory haemodynamics for all configurations, meeting the minimum performance requirements in the international standard ISO5840-3 and an acceptable clinical endpoint^27^ (EOA>1.25 cm^2^ at operating conditions equivalent to a cardiac output of 5 lpm). Diagrams of the EOA, ∆p, and forward flow energy loss for each configuration at the different stroke volumes are represented in figure 3. The results in the entire operating range are extrapolated based on a second order polynomial function (ie, quadratic) fitting. It is evident from the diagrams that there is very little difference for all configuration, with the exception of C3 (aligned, no native leaflets), which displays better performance in terms of ∆p, EOA and energy loss.

**Table 1:** Effective orifice area, transvalvular pressure drop, and systolic energy losses for all configurations at all stroke volumes: mean value, ±SD and percentage value with respect to configuration representing an aligned valve within native leaflets

Global parameter	Stroke volume (mL)	C1	C2	C3	C4
Effective orifice area (cm^2^)	28.6	1.71±0.02	1.98±0.02 *(115%)*	2.36±0.02 *(138%)*	2.03±0.02 *(119%)*
	50.0	2.21±0.02	2.24±0.02 *(101%)*	2.51±0.03 *(114%)*	2.35±0.02 *(106%)*
	71.4	2.35±0.02	2.32±0.02 *(99%)*	2.55±0.03 *(109%)*	2.44±0.02 *(104%)*
	92.9 (86.2 for TA2)	2.33±0.02	2.42±0.02 *(104%)*	2.52±0.03 *(108%)*	2.40±0.02 *(103%)*
Pressure difference (mm Hg)	28.6	3.3±0.08	3.1±0.08 *(94%)*	2.1±0.06 *(63%)*	2.8±0.07 *(85%)*
	50.0	4.8±0.11	4.4±0.10 *(92%)*	3.8±0.09 *(78%)*	4.3±0.10 *(90%)*
	71.4	8.1±0.14	8.2±0.14 *(101%)*	7.1±0.12 *(88%)*	7.8±0.13 *(96%)*
	92.9 (86.2 for TA2)	14.1±0.21	11.9±0.18 *(84%)*	13.5±0.20 *(96%)*	14.7±0.23 *(104%)*
Systolic energy losses (mJ)	28.6	5.4±0.3	5.3±0.2 *(98%)*	7.1±0.3 *(131%)*	8.2±0.4 *(152%)*
	50.0	32.8±0.7	28.1±0.6 *(85%)*	25.8±0.6 *(78%)*	31.7±0.7 *(97%)*
	71.4	82.4±2.0	78.0±1.8 *(95%)*	71.3±1.8 *(87%)*	77.8±1.9 *(94%)*
	92.9 (86.2 for TA2)	203.8±4.8	164.8±4.5 *(71%)*	190.5±5.2 *(93%)*	227.2±5.8 *(111%)*

**Figure 4:**
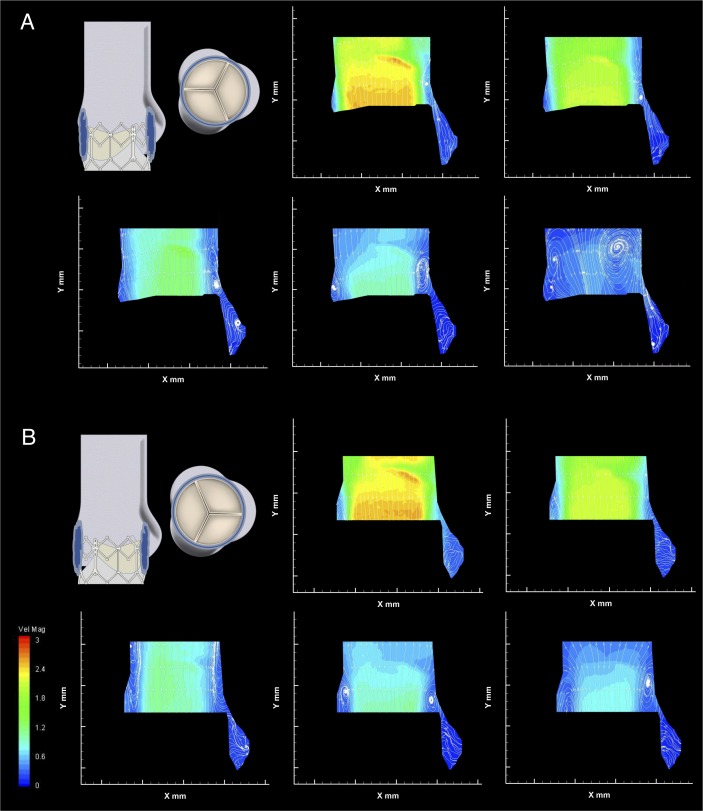
Native leaflet velocity contour maps and streamlines. (A) Configuration C1, aligned valve within native leaflets). (B) Configuration C2, misaligned valve within native leaflets.

**Figure 5:**
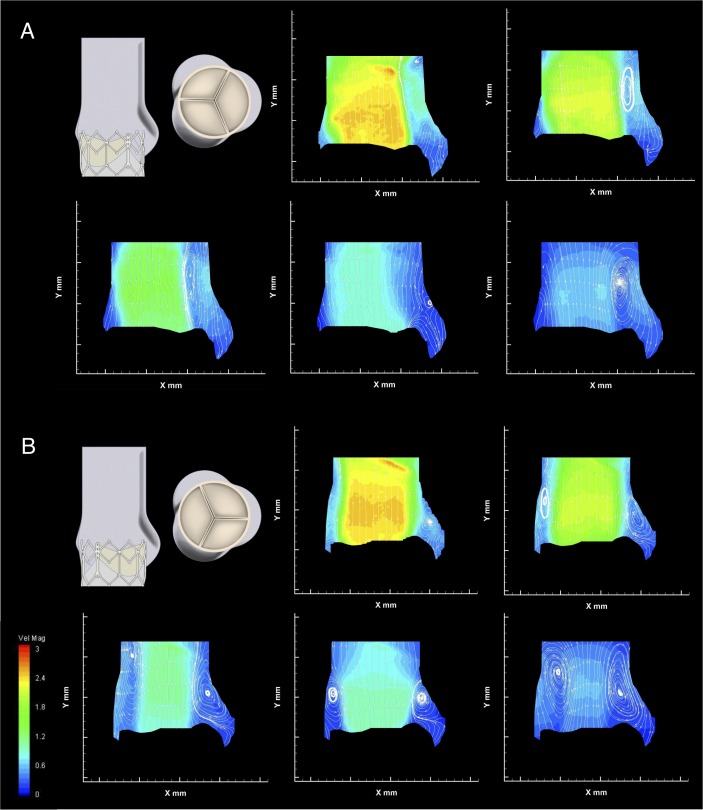
Non-native leaflet velocity contour maps and streamlines. (A) Configuration C3, aligned valve with no native leaflets. (B) Configuration C4, misaligned valve with no native leaflets.

**Figure 6:**
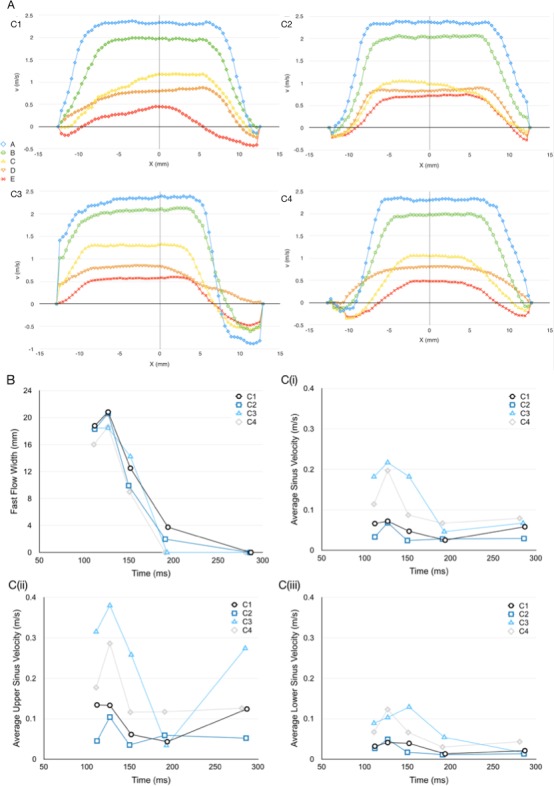
PIV derived data. (A) Downstream velocity across sinotubular junction for the five instants analysed via particle image velocimetry for each configuration. (B) Fast flow width at instants A–E of each configuration. (C) Average velocity within the (i) whole-sinus, (ii) upper-sinus and (iii) lower- sinus for each instant of each configuration.

Figures 4 and 5 display the PIV contour maps of the velocity magnitude and streamlines for each configuration, at the different reference instants, for a stroke volume of 71.4 mL. Figure 6A displays the downstream velocity (*v_STJ_*) at the STJ diameter at each reference instant, for each configuration. These diagrams were used in the calculation of the FFW for each instant, with a collated plot of these values presented in figure 6B. The average magnitude of the velocity vectors within the whole-sinus, the upper-sinus and the lower-sinus for each instant and configuration is shown in figure 6C. PIV data for all configurations at each analysed instant are summarised in table 2.

**Table 2:** Instantaneous data from PIV analysis for all configurations at all instants, with the exception of instant E for the fast flow width (as this was zero for all configurations) and only instant B is presented for the peak velocity

Parameter	Instant	C1	C2	C3	C4
Peak velocity (m/s)	B	2.51%±1.1%	2.59%±1.0%	2.64%±1.1%	2.64%±0.9%
Fast flow width (mm)	A	18.8	18.3	18.5	16.0
	B	20.8	20.6	18.5	18.8
	C	12.5	9.9	14.2	9.0
	D	3.75	2.0	0.0	0.0
Average full sinus velocity (m/s)	A	0.066%±14.6%	0.033%±25.5%	0.182%±5.1%	0.114%±7.4%
	B	0.072%±13.3%	0.068%±12.4%	0.217%±4.3%	0.197%±4.3%
	C	0.047%±20.4%	0.024%±35.0%	0.182%±5.1%	0.087%±9.7%
	D	0.025%±38.4%	0.028%±30.0%	0.046%±20.2%	0.067%±12.5%
	E	0.058%±16.6%	0.029%±29.0%	0.209%±4.5%	0.079%±10.6%
Average upper sinus velocity (m/s)	A	0.134%±7.2%	0.045%±18.7%	0.315%±3.0%	0.177%±4.8%
	B	0.133%±7.2%	0.104%±8.1%	0.380%±2.5%	0.286%±2.9%
	C	0.061%±15.7%	0.035%±24.0%	0.258%±3.6%	0.116%±7.2%
	D	0.043%±22.3%	0.059%±14.2%	0.034%±27.4%	0.117%±7.2%
	E	0.124%±7.7%	0.052%±16.2%	0.274%±3.4%	0.126%±6.7%
Average lower sinus velocity (m/s)	A	0.032%±30.0%	0.027%±31.1%	0.089%±10.5%	0.067%±12.5%
	B	0.041%±23.4%	0.049%±17.1%	0.103%±9.0%	0.123%±6.8%
	C	0.039%±24.6%	0.017%±49.4%	0.129%±7.2%	0.066%±12.7%
	D	0.013%±73.9%	0.011%±76.4%	0.054%±17.2%	0.030%±28.0%
	E	0.021%±45.7%	0.013%±64.6%	0.164%±5.7%	0.043%±19.5%

Velocity data includes PIV velocity uncertainty, as described in the online supplementary material, expressed as a percentage.

The case of perfect alignment of the TAV and native valve commissures (configuration C1) is described in figure 4A. The central jet flow, in early systole, expands after exiting the valve, occupying much of the STJ, extending to the root wall on the commissural side of the valve. The largest FFW is reached during the acceleration (instant A), equal to 20.8 mm. The velocity profile across the STJ has a peak velocity of 2.4 m/s and shows a nearly symmetrical distribution until late systole, with a flat central profile, and the peak velocity in the whole region of investigation is 2.51 m/s, within the threshold for acceptable prosthesis performance.^27^ Throughout systole, the central jet is accompanied by a small vortex, which forms at the exit of the native leaflets, next to the sinus around the level of the STJ. This vortex appears to promote structured flow into the sinus throughout the systole, with an additional vortex apparent in the widest part of the sinus during mid-late systole.

The flow in the sinus suggests washout through the full systolic phase, particularly in the upper-sinus, where return flow in late-systole raises the average velocity back to early-systole levels. The flow on the commissural side of the valve extends to the root wall until late in systole, when a second vortex appears, again positioned at the exit of the native leaflets. This does not seem to affect significantly the central jet width.

Rotating the valve 60° out of phase with the ideal alignment within the native leaflets resulted in little change to the global haemodynamic performance, as shown in figure 3. The central jet speed, peaking at 2.59 m/s, and FFW are also similar to the aligned configuration, although the fluid flow at the edge of this jet is altered. The velocity contour maps in figure 4B show a vortex above the cusp of the prosthetic leaflet throughout systole, which is now aligned with the native commissure, at the expense of the vortex on the sinus side of the valve, which appears only late in systole. This appears to affect the sinus flow in the upper sinus, particularly at peak systole, as shown in figure 6C(ii). Flow velocity in the lower-sinus remains at the low magnitudes measured for configuration C1.

In addition, the central jet flow no longer consistently extends to the root wall on the commissural side of the valve—instead, a region of unstructured slow flow is clearly observed between the root wall and the jet flow throughout systole, with oscillatory features evident due to the downstream translation of vortices along the aortic tract and the changing location of central jet flow extension to the root wall.

Removing the native leaflets from the experimental setup results in a bigger difference between the aligned and misaligned configurations. In the case where the TAV is aligned with the native valve (C3), the Δp and forward flow energy loss are consistently lower for all operating conditions, as seen in figure 3. The central jet is directed closer to the commissural wall, as shown in figure 5A, and though its maximum width is slightly smaller than in previous cases, with a similar peak velocity to the previous configurations of 2.64 m/s, the central jet span is maintained longer during the systolic phase (thus justifying the larger EOA measured through the Gorlin’s equation). On the sinus side, a vortex generates early in systole, much larger than for configuration C1, narrowing the jet flow to the central region of the lumen. Flow in the sinus is much greater than in any other configuration. The flow appears to be clearly defined, providing thorough washout of the sinus throughout systole. There is no evidence of a commissural side vortex at any stage of systole for this configuration.

Misalignment of the valve without native leaflets produces two comparably sized vortices on either side of the valve, present from peak systolic flow, narrowing the FFW as shown in figure 5B, with the same peak velocity of 2.64 m/s detected as in C3. Flow in the sinus, though slower than in C3, is considerably faster than for the configurations including the native leaflets, with well-defined streamlines throughout the cardiac cycle. Similar to the misaligned configuration in C2, a region of unstructured slow flow separates the central jet flow from the commissural side root wall throughout systole, including the transfer of vortices.

## Discussion

Misalignment of a TAV within native leaflets has little effect on global haemodynamic performance parameters such as EOA or Δp. The PIV velocity fields, which provide a thorough description of the flow at specific instants, show that the central fast flow jet properties of the aligned and misaligned configurations are similar, in agreement with these global haemodynamic performance parameters. However, the slower flow outside of this jet does modify its features as the alignment of the valve with the sinuses changes, with misalignment of the TAV resulting in some reduction of the flow velocity within the sinuses, particularly in their upper region, as well as alterations to the flow in the vicinity of the native commissures.

The lack of any significant difference in the central jet flow of the TAV as its alignment with the aortic root is altered may be a result of the presence of the extension of the native leaflets beyond the downstream exit of the TAV. Permanently held open in a pseudocylindrical shape, this relatively uniform geometry may mean the orientation of the TAV has little effect on the resultant central jet flow, with the cusp of this cylinder leading to vortex shedding, as evidenced by the vortices present on both sides of the valve in late systole. This effect may be more pronounced when a TAV is implanted into a native anatomy as opposed to into a preimplanted bioprosthetic surgical valve, as the native leaflets extend further downstream than the prosthetic leaflets.^28^ Similarly, balloon-expandable TAVs, with a typically annular implantation and shorter downstream projection, may be more affected than self-expanding TAVs, which are often implanted in a more supra-annular position with their leaflets operating further downstream in the aortic root.^13^

**Figure 7:**
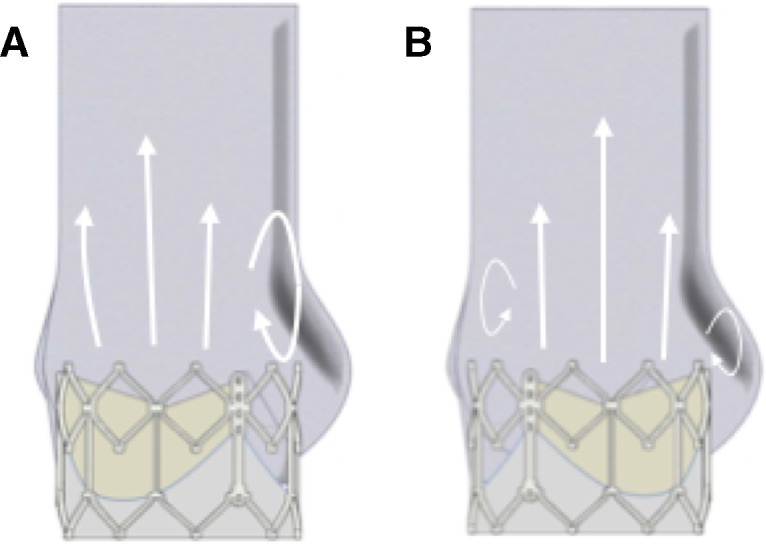
Vortical behaviour changes due to valve alignment. (A) Commissure to commissure alignment results in centre of moving leaflet and maximum sinus bulge being aligned, resulting in a single, larger vortex next to the sinus. (B) Non-alignment of the commissures means the two factors are not aligned, resulting in two weaker vortices forming, one next to the bioprosthetic commissure and one next to the sinus.

However, as the operating leaflets of the TAV have an effect on the location of the circulations at the edge of this jet flow, the strength of the resultant vortices is affected by the orientation of the TAV with respect to the aortic root. Vortical behaviour in the sinus in the physiological root is connected to both vortex shedding from the cusp of the moving leaflet and to the presence of the sinus bulge.^11 29^ When these two factors concur in the same region, as in the aligned configurations, the resultant vortex on the sinus side of the root is stronger, illustrated in figure 7, leading to increased backflow into the sinus and improved washout. Misalignment of the valve, inherently possible with TAV procedures, may reduce either 1 of the 2 factors of vortex generation, producing a weaker vortex in (or next to) the sinus and associated reduced washout. The jet flow does not extend to the root wall, with the unstructured slow flow and translation of vortices outside of this central fast flow reminiscent of both the flow downstream of a stenotic valve and the flow within an ascending aortic dilation.^11^ The resulting blood recirculation and non-physiological vortices next to the wall of the root have been associated with intraluminal thrombosis, leaflet cusp deterioration or aneurysm.^11 30–32^ Also, oscillatory low wall shear stress and flow separation have been linked to plaque formation and enhanced atherogenesis.^33^ In conjunction with the increased stresses experienced by non-aligned TAVs,^12^ it is apparent that, although the haemodynamic performance of the valve is not significantly affected, non-alignment of TAVs might lead to some long-term consequences for the patient and reductions in the prosthetic’s functional life.

Removing the native leaflets from the experimental model resulted in a greater difference between the aligned and misaligned configurations, as the removal of the barrier between the TAV and the sinuses enabled a greater confluence of the two vortical generation factors described above, and flow dynamics more reminiscent of that observed in native and SAV aortic regions. The major disparity of the flow patterns and the improved valve performance obtained between the configurations with and without mock native leaflets clearly indicate that inclusion of a model of native leaflets in testing of TAVs should be introduced in regulatory requirements. In fact, the presence of native leaflets is currently often neglected in both in vitro and in silico studies,^14–16 34^ leading to possibly unrealistically optimistic flow distributions and valve efficiencies.

Though the presence of the native leaflets produces only minor effects on the global valve performance, it appears to severely impair the washout of the sinuses, reducing the peak of average sinus velocity to about 1/3 compared with the configurations without native leaflets. This is associated with regions of stagnation, which may increase the thrombogenicity of the region and provide a possible factor in the reported cases of subclinical leaflet thrombosis in TAVs.^35^ Valve misalignment may further reduce the blood flow in the sinuses, even though the detected difference in the peak average velocity in the sinus is <10% for the worst possible orientation. It can be expected that, for intermediate misalignments, the effect would be even less evident. Although small, any reduction in sinus blood flow may increase the likelihood of coronary obstruction due to thrombus formation, which may occur within 2 months of TAV implantation.^36^ Therapeutic advice for patients who are predisposed towards a higher chance of blood coagulation could be more inclined towards anticoagulation medication if the TAV can be determined to be misaligned after implantation.

The study included some limitations. The interpretation of the results needs to take into consideration that the analysed case is an idealisation, where the model for the native leaflets was at the lower range of aortic leaflet thickness,^37^ and circular deployment of the valve was assumed, despite the fact that the irregularities in the leaflets’ anatomical shape and calcification distribution can lead to asymmetrical expansions.^38^ The consequences of an asymmetrical and non-aligned deployment could be an interesting future study. Moreover, no coronary flows were simulated, which may introduce some degree of asymmetry, and no compliance was modelled in the aortic roots. Though the latter is an approximation, it must be considered that many recipients of TAVs are elderly patients^3^ with lower compliance aortic roots,^23^ and so these findings represent a significant proportion of the patient population. In addition, the fluid used in this investigation was Newtonian, and so some departure from the physiological flow behaviour is to be expected. All these factors might further shade the small difference observed in the wash-out flow associated with the valve alignment.

Finally, no out of plane motion or flow structures can be detected with the PIV setup used, though it is expected that the plane investigated in this study captures the most relevant flow structures.

In conclusion, the presented study indicates that the misalignment of a TAV within native leaflets has negligible effect on the bioprosthesis’ performance, but affects flow patterns by the root wall, especially by the sections of the root in the vicinity of the native commissures, and reduces sinus flow. This suggests that correct alignment may lead to some advantage in terms of sinus flow and reduction of non-physiological vorticity above the native commissures. The design of novel devices improving the rotational control of TAVs, together with the use of advanced medical imaging, such as real-time 3D Computed Tomography-fluoroscopy or 3D transoesophageal echocardiography-fluoroscopy fusion imaging^4 5^ could bear some benefit.

Of course, due to the necessary limitations of the *in vitro* study, recording the orientation of TAVs would be recommendable to providing some clinical support to the presented argument, and identify possible association between instances of subclinical thrombosis and misalignment.

On the contrary, simulations in unrealistic configurations where the native leaflets were not included were characterised by substantially different flow features and significant sensitivity to the valve orientation. This implies that the presence of native-like leaflets is essential in order to determine veridical results, revealing the need for an explicit demand to include this feature in current regulations for preclinical assessment.

10.1136/openhrt-2019-001132.supp1 Supplementary data
