# Correction: Does transcatheter aortic valve alignment matter?

**DOI:** 10.1136/openhrt-2019-001132corr1

**Published:** 2019-12-31

Salmonsmith JA, Ducci A, Burriesci G. Does transcatheter aortic valve alignment matter? *Open Heart* 2019;6:e001132. doi: 10.1136/openhrt-2019-001132

The license type of the paper has changed from CC BY-NC to CC BY.

