# Supplementary material for: Does transcatheter aortic valve alignment matter?
Source: Open Heart. 2019 Nov 21;6(2):e001132. doi: 10.1136/openhrt-2019-001132 (PMC6887501; doi:10.1136/openhrt-2019-001132)
Supplement: Supplementary data [file openhrt-2019-001132supp001.pdf]

Supplementary Material

1 Pulse Duplicator

A Vivitro Superpump System SP3891 (ViVitro Labs Inc., Canada) was used to generate and monitor the required pressure and flowrate through the mock aortic root. Millar Mikro-tip® pressure catheters measured the pressures in the left ventricle and aorta with a resolution of ± 0.001 mmHg, whilst instantaneous volumetric flow rate was acquired via an electromagnetic flowmeter (Carolina Medical Model FM501, Carolina Medical Electronics Inc., USA). A high-speed camera (Sony Cyber-shot DSC-RX100 IV) was used to record the operating valve from a downstream view.
In order to mimic a blood viscosity of 4.0 cP at 37 °C and match the refractive index of the silicone root, a mixture of deionised water (48.54% by mass), glycerol (36.22% by mass), and potassium iodide (15.24% by mass) was used, resulting in a Newtonian liquid with a similar refractive index as the mock aortic root (n = 1.4), and a dynamic viscosity of 4.0 cP at a temperature of 25 °C. A small amount of sodium thiosulphate was added to ensure the fluid remained transparent.
The effective orifice area and transvalvular pressure drop of the different configurations were calculated in compliance with ISO 5840:2013, whilst the energy losses were determined in accordance with Leefe and Gentle[1], using pressures and flows obtained from pulse duplicator data averaged over 10 cycles.
The Transvalvular Pressure Drop (Δp) for each configuration was calculated as follows:

$$\Delta p = \frac{\int_{P1}^{P2} (p_v - p_a) dt}{t_{P2} - t_{P1}}$$

where p_v is the ventricular pressure (mmHg), p_a is the aortic pressure (mmHg), t is the time (s), $P1$ is the beginning of the systolic pressure drop, and $P2$ is the end of the systolic pressure drop.

The Effective Orifice Area (EOA) for each configuration was calculated as follows:

$$EOA = \frac{q_{v_{RMS}}}{51.6 * \sqrt{\frac{\Delta p}{\rho}}}$$

where ρ is the density of the blood analogue fluid (kg/m^3), and $q_{v_{RMS}}$ is the root mean square of the forward flow (ml/s) through the flow meter, defined as:

$$q_{v_{RMS}} = \sqrt{\frac{\int_{F1}^{F2} q_v(t)^2 dt}{t_{F2} - t_{F1}}}$$

where $q_v(t)$ is the instantaneous flow at time t (ml/s), t_{F1} is time of the first positive flow from ventricle to aorta (s), and t_{F2} is the time of the end of positive flow from ventricle to aorta (s).

The Systolic Energy Loss (E_f) for each configuration was calculated as follows:

$$E_f = k_f * \int_{F1}^{F2} \Delta p * \left(q_v(t)\right) dt$$

where k_f is a constant conversion factor of 0.1333, to convert from mmHg*ml to mJ.

Small variations in the assembly of the root and TAV before each test resulted in variable levels of paravalvular leakage through the *in vitro* housing of the TAV. In order to reduce the effect of the different diastolic leakages and produce comparable systolic flows more appropriate for this study, the stroke volume rather than the cardiac output was considered in this investigation. Stroke volumes of 28.6 ml/cycle, 50 ml/cycle, 71.4 ml/cycle, and 92.9 ml/cycle at a heart rate of 70 beats per minute with 35% systolic duration, with a mean aortic pressure of 100 mmHg, were imposed, corresponding to the systolic output for cardiac outputs of 2 lpm, 3.5 lpm, 5 lpm, and 6.5 lpm respectively, assuming no regurgitation or leakage.

2 Particle Image Velocimetry

Neutrally buoyant hollow glass particles (Dantec Dynamics HGS-10, nominal diameter 10 μm) were used to seed the test fluid, and the planar laser field was generated with a dual cavity YAG laser, 70 mJ -x2- at 15 Hz, 532 nm, instrumented with a cylindrical lens producing a 1.5 mm maximum laser thickness. The instantaneous position of the seeding particles was captured with a high-speed camera (TSI PowerView™ Plus 4MP), whilst external triggering was enabled with a LaserPulse™ Synchronizer (Model 610036, TSI Inc., US, with a resolution of 1 ns).

The time interval (Δt) for each image pair varied according to the speed of the particles, and a range of 80-250 μs enabled full identification of slow and fast flow structures.

The commercial software Insight 4G™ (TSI Inc., US) was used to calculate the velocity vectors from the camera images, obtaining a final spatial resolution of 400 μm via utilisation of an adaptive correlation algorithm. These images were then processed via a recursive Nyquist grid, which iteratively shrank the interrogation region from 64×64 to 32×32 pixels, overlapping by 50%, as well as a fast Fourier transform correlation. This produced 150 velocity fields for each cycle defined instant, which were then averaged to produce one final velocity field, corresponding to a standard deviation below 2.6%. Tecplot™ (Tecplot Inc., US) was used to produce streamlines from these velocity vectors.

The velocity measurements calculated via the PIV methodology contain an inherent uncertainty, due to the random error on the determination of the finite interrogation windows within sub-pixel accuracy, and this uncertainty is calculated as the smallest measurable displacement divided by the time spacing at each acquisition point[2]. As the maximum absolute uncertainty of the measured displacement is around 0.1 pixels[2], the velocity uncertainty, v_ϵ, is calculated via:

$$v_\epsilon = \frac{k * 0.1}{\Delta t}$$

where k is the μm to pixel ratio of the camera (varying between configurations and ranging between 21 and 24), and Δt is the time interval between each PIV image pair, as described above, ranging from 80 μs for the fast flow regions at peak systole to 250 μs for the slow flow regions throughout the cycle.

3 Supplementary Material References

1. Leefe SE, Gentle CR. Theoretical evaluation of energy loss methods in the analysis of prosthetic heart valves. *J Biomed Eng*. 1987;9:121–127.
2. Raffel M, Willert CE, Wereley ST, Kompenhans J. Particle Image Velocimetry - A Practical Guide. 2nd ed. Springer; 2007.
